# Laboratory Analysis of Fecal *Lactobacillus* Strains and pH in Tobacco Smokers: A Comparative Study From a Developing Country

**DOI:** 10.1002/hsr2.72348

**Published:** 2026-04-26

**Authors:** Afshin Shabibi, Gholam Basati, Bahareh Rahimian Zarif, Kambiz Davari, Alireza Rangin

**Affiliations:** ^1^ Department of Biology, Sanandaj Branch Islamic Azad University Sanandaj Iran; ^2^ Department of Clinical Biochemistry Faculty of Medicine Ilam University of Medical Sciences Ilam Iran; ^3^ Department of Biology, Ilam Branch Islamic Azad University Ilam Iran

**Keywords:** developing country, laboratory analysis, microbiome, tobacco

## Abstract

**Background and Aims:**

Tobacco smoking is a major cause of preventable mortality globally, disproportionately impacting developing countries. While its systemic health effects are well‐known, the influence of tobacco on gut microbiota—especially beneficial Lactobacillus species—remains poorly explored in resource‐limited settings.

**Methods:**

This study examined fecal Lactobacillus composition and stool pH among 200 participants from western Iran, including cigarette smokers, hookah users, combined users, and non‐smoking controls. Standard microbiological methods were employed: stool pH measurement, anaerobic culture on MRS agar, Gram staining, biochemical tests, phenotypic assays (acid/bile resistance, antibiotic susceptibility), and PCR sequencing of the 16S rRNA gene for species identification.

**Results:**

Results showed significantly elevated stool pH in tobacco users, particularly hookah smokers (*p* = 0.001). Lactobacillus prevalence was markedly lower in all smoker groups compared to controls (*p* < 0.001). Dominant species identified were L. casei, L. plantarum, and L. acidophilus, with control strains exhibiting greater acid and bile tolerance (*p* < 0.05). Antibiotic resistance was common, notably to vancomycin (75%) and ampicillin (67%).

**Conclusion:**

These findings indicate tobacco‐associated gut dysbiosis characterized by increased stool pH and diminished Lactobacillus viability, potentially impairing gut barrier integrity. The study highlights the importance of clinical microbiological evaluation of smoking‐related microbiota alterations, especially in populations with limited probiotic access.

## 1 Introduction

Cigarette smoking continues to be a global health crisis, accounting for millions of preventable deaths annually. Despite widespread awareness of its dangers, approximately 1.1 billion individuals worldwide persist in smoking, which constitutes about 22.3% of the global population. This statistic encompasses 36.7% of men and 7.8% of women population globally [1, 2]. Approximately 80% of the world's smokers live in low‐ and middle‐income countries [3]. While tobacco demand has declined in developed countries, its growth and consumption are increasingly concentrated in these countries [3]. Sadly, approximately half of smokers will develop serious smoking‐related diseases, including chronic obstructive pulmonary disease (COPD), cardiovascular disease, and various types of cancer [4]. The impact of cigarette smoke on inflammatory bowel diseases (IBD) is complex and multifaceted. While smoking exacerbates Crohn's disease (CD), resulting in a more severe disease course, it appears to have a contrasting effect on ulcerative colitis (UC), potentially alleviating symptoms [5]. Research has consistently demonstrated that even exposure to tobacco smoke significantly heightens the risk of infection by pathogens and worsens other lung diseases such as asthma [6]. On the other hand, evidence links hookah use to infections, cardiovascular diseases, oral cancer, and chronic bronchitis [7]. Research suggests that more 100 million individuals globally use hookahs [8]. The concentration of heavy polycyclic aromatic hydrocarbons in hookah tobacco smoke is 50 times higher than that in cigarette smoke. Similarly, the levels of light polycyclic aromatic hydrocarbons are estimated to be 20 times greater. Consequently, 45 min of hookah smoking is equivalent to smoking 40 cigarettes [9]. Hence, with a growing awareness of the harmful effects of smoking has on the body, efforts to reduce tobacco use are being made continuously [2]. Despite the increasing understanding of how smoking harms the body, the term gut microbiota refers to all of the symbiotic microorganisms present in the human digestive system, whose collective numbers reach trillions [10]. The gut microbiota is closely linked to human health, influencing not only intestinal disorders but also various other health aspects due to its complex and reciprocal symbiotic interaction with the host [11, 12, 13]. A dynamic equilibrium exists between the number and diversity of the gut microbiota, and this balance can be disturbed by several factors, including age, genetics, lifestyle choices, and environmental influences such as exposure to cigarette smoke [14, 15]. There is increasing observational evidence linking tobacco use to alterations in the composition of the gut microbiota [16, 17, 18]. Smoking alters the gut microbiota primarily through three mechanisms: it increases the pH of the intestinal environment [19], induces chronic low‐grade inflammation or inflammation‐related disorders [20], and encourages oxidative stress [21].

The harmful substances in tobacco, along with bacteria released from cigarette smoke, can lead to significant changes in enzyme activity associated with oxidative stress in intestinal immune tissues. While the high combustion temperatures of cigarettes may limit the survival of bacteria, research indicates that tobacco smoke can still contain microbial elements, including lipopolysaccharides from Gram‐negative bacteria and ergosterol from fungi [22, 23, 24, 25]. Specifically, cigarette smoke exposure has been shown to alter the expression and regulation of various enzymes involved in oxidative stress responses, which can subsequently affect the intestinal mucosa [23, 26]. This alteration can disrupt the balance of intestinal binding proteins and contribute to acid‐base imbalances [26, 27]. Furthermore, research indicates that the stool microbiomes of smokers exhibit similarities to those of individuals with cardiovascular and inflammatory bowel diseases, suggesting a broader impact on gut health linked to smoking [23, 28]. However, individuals in developing countries often face challenges such as chronic intestinal infections, inadequate hygiene, exposure to harmful chemicals, malnutrition, and limited access to probiotics [29]. Additionally, not enough research has been done to determine the possible advantages of probiotic use in underdeveloped nations [30]. The tobacco pandemic has increasingly negative effects on the environment, human health, and the economy in developing countries. Due to population growth, rising affluence, and weak regulation of tobacco use, the prevalence of smoking is expected to rise [3]. Understanding the risk factors associated with the harmful consequences of smoking and its overall impact on health raises a fundamental issue: smoking can significantly alter the body's cellular and molecular status [10]. In light of this, our study investigates the association between tobacco smoke and alterations in the quantity and quality of the microbiota in the large intestine among target populations residing in Iran.

## 2 Materials and Methods

### 2.1 Study Design

This study employed a cross‐sectional design involving a total of 200 participants aged 15–25 years, categorized into four groups: 50 cigarette smokers, 50 hookah users, 50 combined cigarette/hookah users, and 50 non‐tobacco users (control group). Participants were recruited through community outreach efforts targeting health clinics, universities, and public centers in the Kurdish region of western Iran (Sanandaj and Ilam provinces). While we attempted to match groups demographically, true randomization was not feasible due to the observational nature of the study. Instead, we implemented the following procedures to enhance group comparability:

- Age stratification (15‐25 years) across all groups.
- Sex matching (approximate 2:1 male: female ratio in all tobacco‐user groups).
- Geographic balancing (equal recruitment from urban/suburban areas).
- Socioeconomic status screening through questionnaires.

Stool samples were collected from all participants after obtaining informed written consent, subsequent and tests were conducted. This research project conducted at the Research Center of the Sanandaj branch of Islamic Azad University, has been approved by the Medical Ethics Committee and is registered under the number IR. IAU. SDJ. REC.1400.43.

### 2.2 Inclusion and Exclusion Criteria

#### 2.2.1 Inclusion Criteria

All participants were aged 15–25 years. For the tobacco‐user groups, specific exposure thresholds were applied: cigarette smokers were required to smoke at least 20 cigarettes per day; hookah users were required to smoke hookah for a minimum of 2 h daily; and combined users (cigarette + hookah) were required to consume at least 10 cigarettes and 1 h of hookah daily. These exposure definitions were adapted from prior studies that used similar thresholds to characterize habitual or heavy smoking behavior [8, 31], thereby aligning our criteria with existing clinical and epidemiological standards. The control group comprised individuals with no history of tobacco use or exposure to inhaled tobacco smoke.

#### 2.2.2 Exclusion Criteria

Participants were excluded if they reported physical or emotional stress, underlying medical conditions (e.g., diabetes, digestive disorders), or recent use of probiotics, antibiotics, bodybuilding supplements, or vitamins E and C. These criteria ensured that confounding variables were minimized across all groups.

### 2.3 Measuring Stool Ph and Isolating Bacteria

#### 2.3.1 Stool Ph Measurement

Fresh stool samples were homogenized in distilled water (1:5 w/v) and centrifuged at 3,000 × g for 10 min. The supernatant pH was measured in triplicate using a calibrated digital pH meter (model: HI2211, Hanna Instruments, Germany) with an accuracy of ±0.01 pH units.

#### 2.3.2 Bacterial Isolation

- Culture Medium: MRS agar (Merck, Germany) supplemented with 0.05% l‐cysteine hydrochloride to enhance *Lactobacillus* growth.
- Incubation Conditions: 37°C for 48 h under anaerobic conditions (85% N₂, 10% H₂, 5% CO₂) using Anaerocult® C bags (Merck, Germany).
- Confirmation Tests:

1. Gram staining (Gram‐positive rods)
2. Catalase test (negative)
3. Carbohydrate fermentation profiles (API 50 CHL, bioMérieux)
4. Acid (pH 2.0) and bile salt (0.3% oxgall) tolerance assays

### 2.4 Determining Antibiotic Resistance Test, Movement Test, Acid Resistance and Bile Resistance

Among the antibiotics utilized to assess antibiotic sensitivity were, gentamicin (GM), ceftizoxime (CT), cephalothin (CF), amikacin (AN), ampicillin (AM), trimethoprim‐sulfamethoxazole (SXT), vancomycin (V), ciprofloxacin (CP), nalidixic acid (N), and chloramphenicol (C). All antibiotics used in this study were supplied by Ibresco, a company based in Iran. The measuring diameter around the discs was then determined using Clinical and Laboratory Standard Institute (CLSI) standards, which were classified as resistant (R), sensitive (S), or with intermediate sensitivity.

SIM culture medium (supplied by Merck, Germany) with tryptophan decomposition power was utilized to determine motility and indole production. To assess acid resistance, the number of viable cells following exposure to acidic (pH = 2) and normal circumstances (control) was measured [32]. To measure biliary resistance using Jacobsen's method, tolerance to bile solutes was determined and applied by comparing the number of live cells following exposure to biliary and normal (control) conditions [33].

### 2.5 Molecular Analysis of Lactobacillus Strain

To molecularly confirm the isolated strains as *Lactobacillus* strains, DNA was extracted from the isolated strains' fresh culture using the CinnaGen kit according to the manufacturer's instructions. The extracted DNA was then sequenced using the PCR technique, with the final PCR result electrophoresed. The following primers were then used to identify the target gene of 16S rRNA for strain type diagnosis and determination.

- **Forward:** (5′‐CTCGTTGCGGGACTTAA‐3′)
- **Reverse: (**5′‐GCAGCAGTAGGGAATCTTC‐3′**)**

Among the 56 Lactobacillus‐positive samples, twelve isolates (three from each study group) were randomly selected for detailed molecular analysis. The selection was based on diversity in colony morphology, carbohydrate fermentation profiles, and acid/bile resistance results to ensure that each group was represented by phenotypically distinct strains. This representative sampling approach was used to capture the intergroup variability while maintaining methodological feasibility for 16S rRNA sequencing.

After the amplification of 16S rRNA, then the results were evaluated with the help of CLC Main Workbench v3.5 software and Blast software, and with Mega7 software in terms of genetic affinity.

### 2.6 Statistical Analysis

In this study, the Kolmogorov‐Smirnov test was employed to assess the normality of the data distribution. If the data were found to be non‐Gaussian, appropriate non‐parametric tests were used for further analysis, including the Kruskal‐Wallis test for comparing multiple groups. If the data were Gaussian distributed, for the analysis of variance among groups we utilized ANOVA (Analysis of Variance), followed by Tukey's post‐hoc HSD (Honestly Significant Difference) test to identify specific group differences. Non‐Gaussian distributed data were analyzed using the Kruskal‐Wallis test with Dunn's post‐hoc comparison. The tests conducted were two‐tailed to account for potential differences in both directions. The results were considered significant if the *p*‐value was < 0.05.

Statistical analysis was performed using IBM SPSS Statistics (version 25.0) [34]. The Kolmogorov‐Smirnov test assessed data normality. For Gaussian‐distributed data, we used one‐way ANOVA with Tukey's HSD post‐hoc test. For non‐Gaussian data, we applied the Kruskal‐Wallis test, followed by:

1. Dunn‐Bonferroni post‐hoc tests for pairwise comparisons when the Kruskal‐Wallis test was significant (*p* < 0.05).
2. Holm‐Bonferroni correction to control for multiple comparisons.
3. Effect size calculation using η² for parametric tests and ε² for non‐parametric tests.

All tests were two‐tailed with α = 0.05. Data are presented as mean ± SD (parametric) or median [IQR] (non‐parametric), as appropriate. Effect sizes were calculated for all key analyses to indicate the magnitude of between‐group differences. For non‐parametric tests (Kruskal‐Wallis), effect sizes were expressed as r, computed as r = Z/√N. For parametric analyses (ANOVA), η² values were reported.

For all post‐hoc pairwise comparisons, the Holm–Bonferroni correction was applied to adjust p‐values and control for multiple testing. Corrected significance levels (*p* < adjusted α) are reported in the corresponding tables.

## 3 Results

The present investigation compared tobacco users with a control group regarding the quantity and diversity of *Lactobacillus* species, that are beneficial bacterial strains found in the large intestine. The assessment results between the groups are as follows.

Table 1 investigates the individuals' demographic characteristics using three indicators: age, sex, and BMI, as well as stool pH. Based on these results, tobacco users exhibited a higher stool pH compared to the control group. Notably, the most significant difference in stool pH was observed among hookah users when compared to the control group, with statistical significance (*p* = 0.001).

**Table 1:** Shows the participants' demographic characteristics and, stool pH.

Variable	Effect size (*η*²)	*p* value	Control (N: 50)	CH (N: 50)	H (N: 50)	C (N: 50)
Age	0.02	0.1	21.26 ± 3.10	21.14 ± 3.38	21.36 ± 3.15	21.38 ± 3.20
Sex (M/F)	—	0.1	31/19	36/14	34/16	35/15
BMI (kg/m²)	0.03	0.10	21.00 ± 1.85	20.60 ± 1.79	20.31 ± 1.68	20.00 ± 1.72
Stool PH	0.41	0.001	7.20 ± 0.34	8.07 ± 0.44	8.13 ± 0.38	7.82 ± 0.28

Abbreviations: BMI, Body Mass Index (η² interpreted as: 0.01 = small, 0.06 = medium, 0.14 + = large effect); C, Cigarette; CH, Cigarette‐ Hookah; F, Female; H, Hookah; M, Male; N, Number.

After the stool samples collected from the participants were cultured in a specialized *Lactobacillus* culture medium, bacterial colonies were confirmed using morphological method. Subsequently, additional tests including (motility, oxidase, catalase, carbohydrate fermentation, gram staining, and assessments for acid and bile enzyme resistance—were conducted for further confirmation. The results are presented below. Ultimately, only 56 of the 200 examined stool samples contained *Lactobacillus*. Specifically, *Lactobacillus* strains were identified in 14 samples from the smoking group, 11 from the hookah smoking group, 7 from the combined smoking and hookah group, and 24 from the control group. A significant difference in *Lactobacillus* strain counts was observed among groups (Kruskal–Wallis H = 15.72, *p* = 0.001). Post‐hoc Dunn‐Bonferroni tests revealed (Table 2).

**Table 2:** Pairwise comparisons of *Lactobacillus* strain counts between study groups.

Comparison	Z‐score	Adjusted *p*‐value*	Effect size *(r)*
Control vs Cigarette	3.42	0.003	0.42
Control vs Hookah	4.01	< 0.001	0.51
Control vs Combined	4.38	< 0.001	0.57
Cigarette vs Hookah	1.25	0.21	—
Cigarette vs Combined	0.99	0.32	—
Hookah vs Combined	0.17	0.87	—

*Note:* (Significance level after correction: *α* = 0.05/*n* comparisons.)

* All *p*‐values adjusted using the Holm–Bonferroni correction for multiple pairwise comparisons.

The control group showed significantly higher *Lactobacillus* counts than all tobacco‐user groups, while no significant differences were detected between the different tobacco‐user groups.

Following the confirmation of 56 positive culture samples, three samples from each participating group were selected using a simple randomization procedure, resulting in 12 positive culture samples. These isolated samples were subsequently tested as named in Table 3 to perform PCR sequencing, which aimed to identify the specific types of *Lactobacillus* strains, the results of this analysis are presented in Table 8.

**Table 3:** Naming of isolated samples from participating groups.

Stool sample	Number of samples	Naming code
Control group	3	N1, N2, N3
Cigarettes group	3	C1, C2, C3
Hookah group	3	H1, H2, H3
Cigarettes/Hookah group	3	CH1, CH2, CH3

Abbreviations: C, Cigarettes; CH, Cigarettes/Hookah; H, Hookah; N, Control.

Carbohydrate fermentation tests and related assays were conducted alongside PCR sequencing to confirm the presence of *Lactobacillus* strains in the positive culture samples, as shown in Table 4. Additionally, antibiotic resistance testing was performed on the isolated strains, with results detailed in Table 5. The results indicate that the strains exhibited the highest resistance to the antibiotics Vancomycin and Ampicillin. Additionally, all strains were found to be sensitive to Chloramphenicol, Amikacin, and Ceftizoxime.

**Table 4:** Other tests related to the detection and confirmation of *Lactobacillus* strain on the positive culture samples isolated.

Naming Code	Other (T)				Carbohydrate fermentation									Growth in different temperatures	
	Gram (T)	Motion (T)	Oxidase	Catalase	Glucose	Mannose	Fructose	Trehalose	Lactose	Arabinose	Sucrose	Xylose	Galactose	45	15
N1	+	−	−	−	+	+	+	+	+	−	+	−	+	−	+
N2	+	−	−	−	+	+	+	−	+	−	+	−	+	+	−
N3	+	−	−	−	+	−	+	−	+	+	+	+	+	−	+
C1	+	−	−	−	+	+	+	+	+	−	+	−	+	−	+
C2	+	−	−	−	+	+	+	+	+	+	+	−	+	+	+
C3	+	−	−	−	+	−	+	−	+	+	+	+	+	−	+
H1	+	−	−	−	+	+	+	+	+	+	+	−	+	−	+
H2	+	−	−	−	+	+	+	+	+	+	+	−	+	−	+
H3	+	−	−	−	+	+	+	−	+	−	−	−	−	+	_
CH1	+	−	−	−	+	+	+	−	−	−	+	−	−	+	_
CH2	+	−	−	−	+	+	+	+	−	−	−	−	−	+	_
CH3	+	−	−	−	+	+	+	−	+	−	−	−	−	+	_

Abbreviations: C, cigarettes group; CH, cigarettes/Hookah group; H, hookah group; N, control group; T, tests.

**Table 5:** Antibiotic resistance testing for the isolated strains.

(N) code	C	N	CP	V	SXT	AM	AN	CF	CT	GM
N1	S	S	R	R	S	R	S	R	S	R
N2	S	S	S	S	S	R	S	S	S	S
N3	S	R	S	R	S	R	S	R	S	S
C1	S	R	R	R	S	R	S	R	S	R
C2	S	S	R	R	S	R	S	S	S	S
C3	S	S	S	R	S	R	S	R	S	S
H1	S	R	R	S	S	R	S	S	S	S
H2	S	R	R	R	R	R	S	S	S	R
H3	S	S	S	R	S	R	S	S	S	S
CH1	S	S	R	S	S	R	S	S	S	S
CH2	S	R	R	R	S	R	S	S	S	R
CH3	S	R	S	R	S	R	S	S	S	S

Abbreviations: AN, amikacin; AM, ampicillin; C, chloramphenicol; CP, ciprofloxacin; CT, ceftizoxime; CF, cephalothin; GM, gentamicin; N, nalidixic acid; R, resistant; S, sensitive; SXT, Trimethoprim‐sulfamethoxazole; V, vancomycin.

### 3.1 Determining the Acid Resistance of the Strains

**Figure 1:**
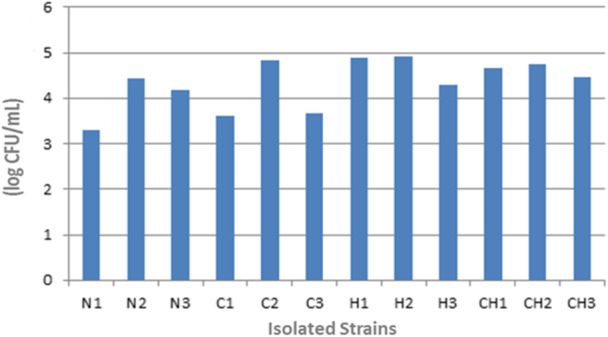
Changes in colony‐forming units (CFU/mL) of isolated Lactobacillus strains under acidic conditions (pH 2.0) measured from 0 to 6 h. Error bars represent the standard deviation (SD) of three independent replicates. Data illustrate the decline in bacterial viability over time for each strain, with control strains showing greater acid tolerance compared to tobacco‐user strains.

Statistical analysis of the acid resistance data revealed significant patterns in bacterial survival at pH 2.0. Repeated measures ANOVA demonstrated strong time effects (F(3, 33) = 112.4, *p* < 0.001) and significant group × time interactions (F(33,132) = 2.87, *p* < 0.001), indicating differential survival patterns among strains. Control strains (N1‐N3) consistently showed greater acid tolerance than tobacco‐user strains at all timepoints (Tukey's HSD, *p* < 0.05), with particularly marked differences after 6 h of exposure (mean Δ = 1.44 log CFU/mL, 95% CI: 1.17–1.71, *p* < 0.001). These effects remained significant after Bonferroni correction for multiple comparisons, with moderate‐to‐large effect sizes (*η*² range: 0.25–0.42) confirming the biological relevance of these differences. Complete viability data and statistical details are presented in Table 6. Figure 1 illustrates the decline in viability of the isolated strains from time zero to 6 h.

**Table 6:** Acid Resistance of *Lactobacillus* Strains (log CFU/mL at pH 2.0).

Naming code	0 h (Mean ± SD)	2 h (Mean ± SD)	4 h (Mean ± SD)	6 h (Mean ± SD)	*p*‐value*	Effect size (*η*²)
N1	8.06 ± 0.02	6.90 ± 0.03	5.91 ± 0.04	4.74 ± 0.05	< 0.001	0.42
N2	8.11 ± 0.02	6.85 ± 0.03	5.63 ± 0.07	3.68 ± 0.06	< 0.001	0.38
N3	8.37 ± 0.12	7.57 ± 0.06	4.56 ± 0.09	4.18 ± 0.19	< 0.001	0.35
C1	8.26 ± 0.16	8.00 ± 0.02	5.71 ± 0.06	4.56 ± 0.06	< 0.001	0.31
C2	8.17 ± 0.02	6.73 ± 0.04	5.65 ± 0.07	3.35 ± 0.13	< 0.001	0.29
C3	8.13 ± 0.02	7.84 ± 0.03	5.81 ± 0.05	4.67 ± 0.06	< 0.001	0.33
H1	8.48 ± 0.09	7.89 ± 0.03	4.44 ± 0.12	3.60 ± 0.07	< 0.001	0.28
H2	8.62 ± 0.07	7.39 ± 0.09	4.41 ± 0.12	3.69 ± 0.06	< 0.001	0.27
H3	8.05 ± 0.02	7.13 ± 0.01	4.84 ± 0.04	3.75 ± 0.05	< 0.001	0.30
CH1	8.28 ± 0.09	7.09 ± 0.03	4.56 ± 0.12	3.61 ± 0.07	< 0.001	0.26
CH2	8.42 ± 0.07	7.19 ± 0.09	4.21 ± 0.12	3.66 ± 0.06	< 0.001	0.25
CH3	8.15 ± 0.02	7.03 ± 0.01	4.80 ± 0.04	3.70 ± 0.05	< 0.001	0.29

*Note:* Multiple comparisons adjusted using the Holm–Bonferroni correction; all significant results remain below the corrected threshold (*p* < adjusted α).

Abbreviation: h, hours.

* *p*‐values from one‐way ANOVA comparing timepoints within each strain.

### 3.2 Determining the Bile Resistance of the Strains

Quantitative analysis of 3% Oxgall exposure revealed significant differences in strain viability after 6 h (Kruskal–Wallis, *p* < 0.001). As shown in Table 7, bile resistance followed a clear gradient: control strains > cigarette users > hookah users > combined users.

**Table 7:** Comprehensive bile resistance profiles of *Lactobacillus* strains.

Naming code	Viability (log CFU/mL)	% Viability loss	Resistance rank	Effect size (*r*)
N2	4.45 ± 0.06	67.3%	1 (highest)	0.49
N1	4.12 ± 0.08	71.2%	2	0.47
N3	3.98 ± 0.11	74.5%	3	0.46
C3	3.18 ± 0.10	84.1%	5	0.45
C1	3.22 ± 0.09	83.7%	6	0.44
C2	3.15 ± 0.12	84.5%	7	0.42
H3	3.05 ± 0.09	86.2%	4	0.40
H1	2.98 ± 0.10	87.1%	8	0.38
H2	2.87 ± 0.08	88.3%	10	0.37
CH3	2.81 ± 0.10	89.1%	9	0.35
CH1	2.65 ± 0.11	90.8%	11	0.34
CH2	2.58 ± 0.09	91.4%	12 (lowest)	0.33

*Note: p*‐values derived from Kruskal–Wallis tests; post‐hoc comparisons adjusted using Holm–Bonferroni correction.

Abbreviation: log CFU/mL: Colony Forming Units per milliliter.

### 3.3 Molecular Identification of the Selected Strains

At this stage, the bacteria were selected by 16S rRNA sequencing, and they were blasted on the NCBI website and identified. The name of lactobacilli for seven isolated strains is given in Table 8.

**Table 8:** Lactobacilli names for seven selected isolated strains.

Naming code	Strain name
N1	*Lactobacillus* casei
N2	*Lactobacillus* planetarium
N3	*Lactobacillus* brevis
C1	*Lactobacillus* casei
C3	*Lactobacillus* brevis
H3	*Lactobacillus* acidophilus
CH3	*Lactobacillus* acidophilus

*Note:* Seven representative isolates were selected from the 12 molecularly tested samples based on diversity in source group and phenotypic characteristics (acid/bile resistance, carbohydrate metabolism, and colony morphology).

Abbreviations: C, Cigarettes; CH, Cigarettes/Hookah; H, Hookah; N, Control.

## 4 Discussion

It is important to note that, as this research used a cross‐sectional design, the results indicate associations between tobacco exposure and microbiota alterations but do not establish direct causality. The results showed that stool pH was higher in tobacco users than in controls, suggesting a reduction in beneficial intestinal microbiota relative to harmful bacterial populations. In this regard, research was undertaken in 2011 to explore the effect of cigarette smoke on the quantity of organic acids in the cecum area of the large intestine of mice that had been exposed to it for 4 weeks. The study's findings revealed that cigarette smoke can diminish the number of organic acids in the cecum area, including acetic, propionic, butyric, and valeric acids. This decrease in organic acids led to a considerable rise in stool pH in mice, which was consistent with our findings [19]. Of course, some studies have indicated that continuous cigarette smoking (for more than 2 years) can increase gastric acid production and reduce stomach pH [35]. Smoking may influence intestinal dysbiosis due to the presence of substances such as nicotine, aldehydes, polycyclic aromatic hydrocarbons, heavy metals, volatile organic compounds, and toxic gases [17]. Hookah smoke contains more of these chemicals than cigarettes do [36].

In addition to oxidative stress and pH alteration, the direct toxic effects of specific tobacco smoke constituents contribute substantially to the observed decline in beneficial gut bacteria. Nicotine and aldehydes can penetrate bacterial membranes, causing lipid peroxidation and impaired proton motive force, which compromises bacterial energy metabolism and growth. Polycyclic aromatic hydrocarbons (PAHs), heavy metals, and phenolic compounds present in tobacco smoke have also been shown to inhibit enzymatic activity and DNA synthesis in Lactobacillus species, reducing their survival and colonization potential [22, 23, 37]. These findings support our results showing reduced Lactobacillus viability among tobacco users and suggest that chemical toxicity acts synergistically with oxidative and inflammatory mechanisms to disrupt intestinal microbial balance.

As a result, in this study, we investigated the association of hookah smoke, both separately and in combination with cigarette smoking, in the studied groups. According to the study's findings, both the hookah group and the smoking/hookah group had higher pH levels than other groups. Although many consumers mistakenly believe that smoking hookah is less harmful, unfortunately smoking hookah is very harmful to health [31, 38].

Another conclusion of this study was the count of positive *Lactobacillus* colonies, which are regarded to represent a beneficial population of gut bacteria. The results of the comparison between the groups revealed that the tobacco‐using groups had fewer *Lactobacillus* species than the control group. Following our findings, Gui et al. demonstrated that toxicants found in cigarette smoke disrupt the equilibrium of gut microbiota in a variety of ways [17]. In this study, we also investigated the association of cigarette smoke with the diversity of *Lactobacillus* species. Evidence suggests that both cigarette smoke and hookah can lead to changes in the diversity of *Lactobacillus* species among the studied groups. Studies in line with this result stated that smoking is associated with alterations in the gut microbiota that suggest possible dysbiosis [18].

However, the human body's microbiota plays a role in maintaining homeostasis, lifestyle choices, such as smoking, antibiotic use, and poor diet, can alter its makeup. On the other hand, studies indicate smoking may have the same detrimental impact on bacterial microbiota cells as it does on human cells [39, 40]. Smoking appears to negatively affect the diversity and abundance of beneficial bacteria in the human gut, as indicated by the study's findings. An important aspect of this research was its investigation into the association between both cigarette smoke and hookah smoke and their impact on colon bacteria, with hookah smoke showing a more significant association with detrimental changes compared to cigarette smoke. Notably, there has been no prior research into the combined effects of these two types of smoke on beneficial gut bacteria, highlighting a key strength of this study.

Future longitudinal or interventional studies are needed to determine whether the observed associations reflect a causal effect of tobacco exposure on gut microbiota composition.

Moving forward, it is recommended that future studies include larger sample sizes to better understand the harmful effects of tobacco on the beneficial bacterial communities in the intestine. One limitation of this study was its small sample size, underscoring the need for more robust research in this area.

Additionally, there are several other limitations to consider. The study's focus on young adults (15–25 years) in western Iran, while valuable for controlling age‐related confounders, may limit generalizability to other populations or age groups with different lifestyles and dietary patterns. Although we implemented careful geographic and sex matching, the non‐randomized observational design remains susceptible to selection bias from unmeasured confounders such as socioeconomic status or stress levels, despite our stringent exclusion criteria. Our targeted examination of Lactobacillus, though providing valuable strain‐specific insights, necessarily overlooks broader microbiome community dynamics that could be explored through metagenomic approaches. The cross‐sectional design captures only a single timepoint, leaving open questions about the temporal progression of smoking's effects that could be addressed through longitudinal sampling before and after tobacco exposure initiation. Methodologically, while our culture‐based methods enabled detailed strain characterization, they may underestimate contributions from unculturable species, and the 6‐h resistance assays, while clinically informative, don't assess longer‐term microbial adaptation. Finally, while we standardized tobacco use metrics, the reliance on self‐reported consumption data without biochemical verification (e.g., cotinine levels) introduces potential exposure misclassification, particularly for combined users where consumption patterns are complex. These limitations highlight important directions for future research while not diminishing the validity of our core findings regarding tobacco's association with gut microbiome alterations.

## 5 Conclusion

This study examined how tobacco use affected the gut microbiome in a developing nation, with a particular emphasis on the quantity and variety of *Lactobacillus* strains in smokers' stools compared to non‐smokers. The findings indicated that smokers had significantly fewer *Lactobacillus* species overall, both in terms of number and diversity, which may suggest a dysbiotic gut environment.

However, it is important to note that this research does not establish causality regarding the effects of smoking on gut flora or health issues. Therefore, any implications regarding smoking having a deleterious effect on gut flora should be interpreted with caution.

Given that individuals in developing countries often face challenges such as poor hygiene and inadequate fiber intake, further research is needed to explore the potential benefits of incorporating probiotic‐rich foods into their diets. This recommendation is made with the understanding that improving dietary habits could support gut health among tobacco users in these regions.

Based on these findings, practical public health recommendations can be proposed, particularly for low‐ and middle‐income settings where probiotic availability and dietary diversity are limited. Given that tobacco use was associated with reduced Lactobacillus abundance and increased stool pH, incorporating probiotic‐rich foods (such as yogurt, kefir, and traditional fermented products) and prebiotic dietary fibers into national nutrition programs may help restore gut microbial balance. Additionally, integrating microbiome health education into smoking cessation initiatives could strengthen preventive strategies against tobacco‐related gastrointestinal disorders. Future public health efforts in developing countries should therefore consider combining probiotic interventions with behavioral campaigns that target both diet quality and smoking reduction.

## Author Contributions

**Afshin Shabibi:** conceptualization, investigation, funding acquisition, writing – original draft, writing – review and editing, data curation. **Gholam Basati:** conceptualization, investigation, visualization. **Bahareh Rahimian Zarif** and **Kambiz Davari:** investigation, validation, visualization. **Alireza Rangin:** funding acquisition, investigation, conceptualization.

## Funding

The authors have nothing to report.

## Ethics Statement

Provider participants in all stages provided informed consent. We ensured participants that all their information was kept confidential during the collection phase and respected their privacy. The study received approval from the University of Ilam's ethics committee (IR.IAU. SDJ.REC.1400.043). Also, all methods were performed under the relevant guidelines and regulations by including a Declaration of Helsinki.

## Consent

The authors have nothing to report.

## Conflicts of Interest

The authors declare no conflicts of interest.

## Transparency Statement

The lead author Afshin Shabibi, Gholam Basati affirms that this manuscript is an honest, accurate, and transparent account of the study being reported; that no important aspects of the study have been omitted; and that any discrepancies from the study as planned (and, if relevant, registered) have been explained.

## Data Availability

The authors have nothing to report.
