# Correction To: “Laboratory Analysis of Fecal *Lactobacillus* Strains and pH in Tobacco Smokers: A Comparative Study From a Developing Country”

**DOI:** 10.1002/hsr2.72537

**Published:** 2026-05-17

1

A. Shabibi, G. Basati, B. R. Zarif, K. Davari, and A. Rangin, “Laboratory Analysis of Fecal Lactobacillus Strains and pH in Tobacco Smokers: A Comparative Study From a Developing Country,” *Health Science Reports* 9 (2026): e72348, https://doi.org/10.1002/hsr2.72348.

In the published article, the affiliations (1) and (3) were listed incorrectly as “Department of Biology, Sanandaj Branch, Islamic Azad University, Sanandaj, Iran” and “Department of Biology, Ilam Branch, Islamic Azad University, Ilam, Iran.”

Corrected affiliations should be as follows:

“Department of Biology, Sa.C., Islamic Azad University, Sanandaj, Iran” and “Department of Biology, Il.C., Islamic Azad University, Ilam, Iran”

The online version of this article has been corrected accordingly.

We apologize for these errors.

